# Endoscopic Stenting as Bridge to Surgery versus Emergency Resection for Left-Sided Malignant Colorectal Obstruction: An Updated Meta-Analysis

**DOI:** 10.1155/2017/2863272

**Published:** 2017-07-05

**Authors:** Niccolò Allievi, Marco Ceresoli, Paola Fugazzola, Giulia Montori, Federico Coccolini, Luca Ansaloni

**Affiliations:** 1st Surgical Department, Papa Giovanni XXIII Hospital, Bergamo, Italy

## Abstract

**Introduction:**

Emergency resection represents the traditional treatment for left-sided malignant obstruction. However, the placement of self-expanding metallic stents and delayed surgery has been proposed as an alternative approach. The aim of the current meta-analysis was to review the available evidence, with particular interest for the short-term outcomes, including a recent multicentre RCT.

**Methods:**

We considered randomized controlled trials comparing stenting as a bridge to surgery and emergency surgery for the management of left-sided malignant large bowel obstruction, performing a systematic review in MEDLINE, PubMed database, and the Cochrane libraries.

**Results:**

We initially identified a total of 2543 studies. After the elimination of duplicates and the screening of titles and abstracts, seven studies, for a total of 448 patients, were considered. The current meta-analysis revealed no difference in the mortality rate between the stent group and the emergency surgery group; the postoperative complication rate (37.84% versus 54.87%, *P* = 0.02), the stoma rate (28.8% versus 46.02%, *P* < 0.0001), and the incidence of wound infection (8.11% versus 15.49%, *P* = 0.01) were reduced after stent as a bridge to surgery.

**Conclusion:**

Colonic stenting as a bridge to surgery appears to be a safe approach to malignant large bowel obstruction. Possible advantages of this treatment can be identified in a reduced incidence of postoperative complications and a lower stoma rate. Further RCTs considering long-term outcomes and cost-effectiveness analysis are needed.

## 1. Introduction

Malignant large bowel obstruction (MBO) occurs in 15% to 30% of the patients presenting with colorectal malignancy [1, 2] and is associated with considerable mortality rate (15–20%) and morbidity rate (40–50%) [2, 3].

Colonic stenting was proposed by Dohmoto in 1991 as a palliative option to obtain symptom relief and reduce the need of surgery for stoma creation [4]. Tejero et al. first described the use of self-expanding metallic stents as a bridge to surgery in the management of potentially resectable colorectal cancer presenting with obstruction, allowing bowel decompression and elective surgical planning [5].

The 90-day mortality appears to be higher after urgency/emergency surgery (12.3%), as compared to elective surgical management for colorectal cancer (2.1%) [6]. This justified the conduction of several randomized controlled trials [7–13] comparing the uses of self-expanding metallic stents (SEMS) as a bridge to surgery (SBTS) and emergency surgery (ES) for the management of MBO. The rationale underpinning the use of metallic stents as a bridge to surgery resides in the possibility of bowel decompression and subsequent medical stabilization of the patient, including adequate management of comorbidities and correction of hydroelectrolyte unbalances, and staging of the disease.

The insertion of SEMS has been presented by the World Society of Emergency Surgery as a valuable option in Centres where clinical and technical expertise is available [14]. This technique appears to be safe and effective: recent meta-analyses have shown favourable short-term outcomes using SBTS, as opposed to emergency surgery [15–17].

The purpose of the current study was to compare stenting as a bridge to surgery and emergency surgery for the management of malignant left-sided colonic obstruction, in terms of short-term outcomes. In particular, the aim of the meta-analysis was to update the current evidence in this field, including a recently concluded multicentre randomized controlled trial by Arezzo et al. [8].

## 2. Methods

Methodology for the current meta-analysis was developed from the guidelines of the Cochrane Handbook for Systematic Reviews of Interventions (version 5.1.0) [18] and the Preferred Reporting Items for Systematic Reviews and Meta-Analyses (PRISMA) statement [19].

### 2.1. Literature Search and Study Selection

**Figure 1:**
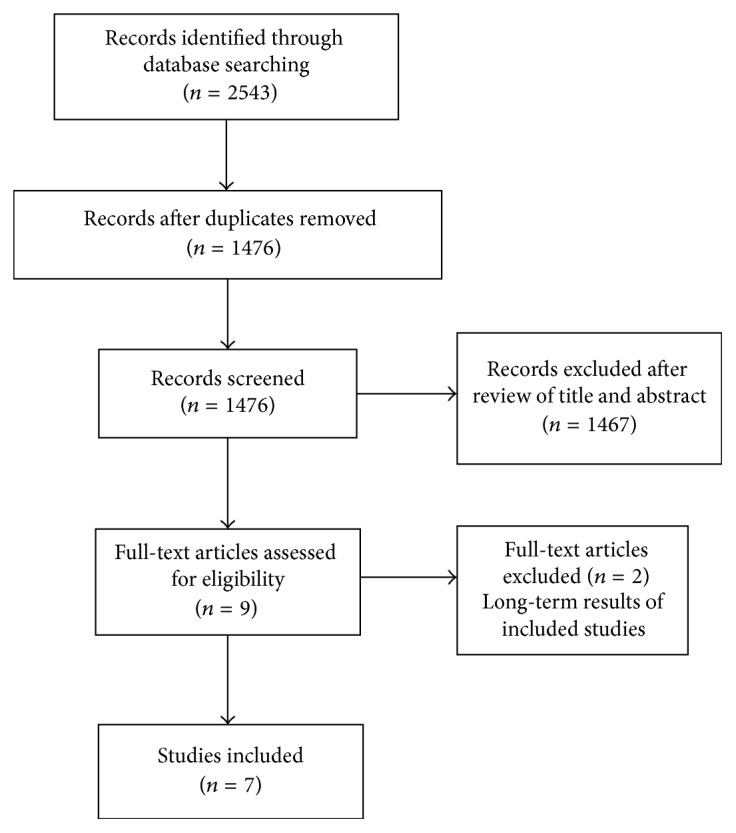
Flow chart of study selection according to PRISMA guidelines.

A Literature systematic search was performed using MEDLINE, PubMed database, and the Cochrane library database for relevant randomized controlled trials comparing SBTS and emergency surgery from January 1990 and December 2016 (Figure 1).

The following search terms were used:* colonic obstruction* or* large bowel obstruction*,* stent* or* colorectal stent,* or* bridge*. With the aim of reducing selection bias, a parallel manual selection was performed using Google Scholar. References from included trials and previous meta-analyses were also screened.

### 2.2. Inclusion and Exclusion Criteria

For the purposes of the current meta-analysis, we considered randomized controlled trials (RCTs) which compared stenting as a bridge to surgery and emergency surgery for left colonic and rectal malignant obstruction in adult patients. No limitations relative to sample size or language were applied.

Exclusion criteria were nonrandomized trials, use of SEMS for palliative treatment, anatomical location of the obstruction other than left colon and rectum, and nonmalignant large bowel obstruction.

### 2.3. Data Extraction

A data sheet was created and data extraction relative to the outcomes of interest was performed by two authors (NA and MC) independently. Differences and disagreements were resolved with a discussion between the two cited authors; in case no agreement could be reached, a diriment decision would have been made by a third author. For the purposes of the meta-analysis, the following data were extracted from each included study (Table 1): year of publication, tumor site, number of patients included, type of intervention, type of SEMS used, time from stenting to surgery, technical and clinical success of stent placement, and data relative to primary and secondary outcomes as described in Table 1.

### 2.4. Primary and Secondary Outcomes

We considered the following primary outcomes for the two different treatment groups: mortality, postoperative complications, primary anastomosis, and stoma rate (Table 2).

The rate of anastomotic leak and that of different infectious complications were recorded as secondary outcomes for the current meta-analysis.

Furthermore, within the SBTS group, we considered data regarding technical success, clinical success, and stent-related complications.

### 2.5. Statistical Analysis

The statistical analysis was performed in compliance with the PRISMA Statement and the Cochrane Handbook for systematic reviews [18]. The statistical software Review Manager Version 5.0 (Copenhagen: The Nordic Cochrane Centre, The Cochrane Collaboration, 2008) was used for the statistical analysis. For categorical values results were reported as weighted risk ratio (RR) and 95% confidence interval, while continuous variables were expressed as weighted mean difference and 95% confidence interval. Heterogeneity was calculated using the *χ*^2^ test; significance was set at *P* < 0.10 and quantified by using *I*^2^. A value of *I*^2^ > 50% indicated significant heterogeneity and prompted the use of random-effect model. With low heterogeneity results were calculated with the fixed-effect model [20, 21].

### 2.6. Assessment of Risk of Bias

Two authors (NA and MC) independently assessed the risk of bias (Table 3) using the Cochrane Collaboration's tool [18] for assessing the risk of bias derived from random sequence generation, allocation concealment, blinding of participants, blinding of outcome assessment, assessment of incomplete data outcome, selective reporting, and other sources of bias.

## 3. Results

Seven randomized controlled trials, published between 2009 and 2016, were included in the current meta-analysis. The main findings for each study are summarized in Table 4.

Inclusion criteria for the considered studies were as follows: adult patients (aged over 18 years at diagnosis) being treated for malignant obstruction of the large bowel distal to the splenic flexure; the diagnosis was confirmed radiologically. Exclusion criteria were as follows: obstruction at anatomical site proximal to the left colonic flexure or with nonmalignant etiology and signs of peritonitis or perforation at admission.

In total, 448 patients were included for the purposes of the meta-analysis: 222 in the SBTS group and 226 in the ES group.

### 3.1. Primary Outcomes

#### 3.1.1. In-Hospital Mortality

**Figure 2:**
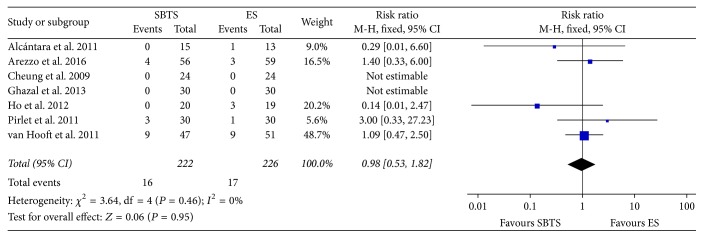
Meta-analysis of mortality rates using fixed-effect Mantel-Haenszel models. Risk ratio shown with 95% confidence intervals. ES: emergency surgery; SBTS: stent as a bridge to surgery.

The mortality rates were reported in all 7 considered studies (Figure 2) and were equal to 7.2% (16 of 222 patients) and 7.52% (17 of 226) within the SBTS group and ES group, respectively. The difference was found to be nonsignificant at meta-analysis (RR 0.98, 95% CI 0.53 to 1.82, *P* value 0.95) and there was no heterogeneity (*I*^2^ = 0%).

#### 3.1.2. Postoperative Complication

**Figure 3:**
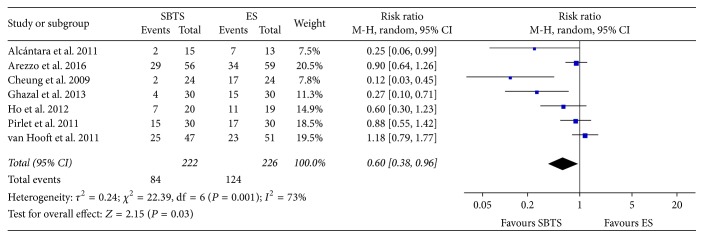
Meta-analysis of postoperative complication rates using random-effect Mantel-Haenszel models. Risk ratio shown with 95% confidence intervals. ES: emergency surgery; SBTS: stent as a bridge to surgery.

Overall, the incidence of postoperative complications (Figure 3) was 37.84% (84 of 222 cases) in the SBTS group, as compared to 124 of 226 patients (54.87%) in the ES group. This difference was statistically significant (RR 0.6, 95% CI 0.38 to 0.96, *P* value 0.02). Heterogeneity was found to be significantly high (*I*^2^ = 73%); therefore random-effect model was considered.

#### 3.1.3. Primary Anastomosis

**Figure 4:**
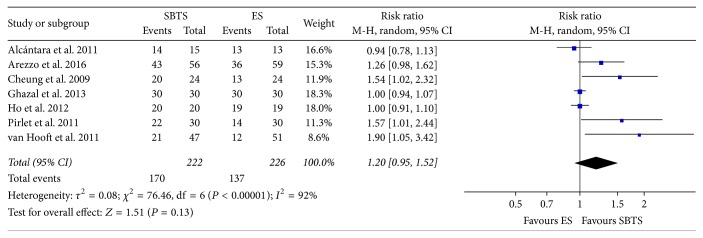
Meta-analysis of primary anastomosis rates using random-effect Mantel-Haenszel models. Risk ratio shown with 95% confidence intervals. ES: emergency surgery; SBTS: stent as a bridge to surgery.

Primary anastomosis rate was reported in order to investigate whether the use of a metallic stent as a bridge to surgery increased the likelihood of receiving a one-stage procedure. Meta-analysis of this outcome (Figure 4) showed an increment in the rate of primary anastomosis in the SBTS group (76.58% for the SBTS group versus 60.62% for the ES group), but this did not reach statistical significance (RR 1.2, 95% CI 0.95 to 1.52, *P* value 0.13). High heterogeneity was evident for this outcome, with *I*^2^ = 92%.

**Figure 5:**
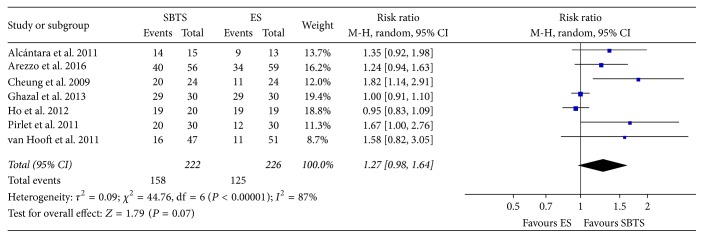
Meta-analysis of successful primary anastomosis rates using random-effect Mantel-Haenszel models. Risk ratio shown with 95% confidence intervals. ES: emergency surgery; SBTS: stent as a bridge to surgery.

Successful primary anastomosis (Figure 5) was achieved in 158 out of 222 cases within the SBTS group (71.17%), as compared to 125 cases out of 226 (55.3%) within the ES group; these findings were not statistically significant (RR 1.27, 95% CI 0.98 to 1.64, *P* value 0.07).

#### 3.1.4. Stoma Rate

**Figure 6:**
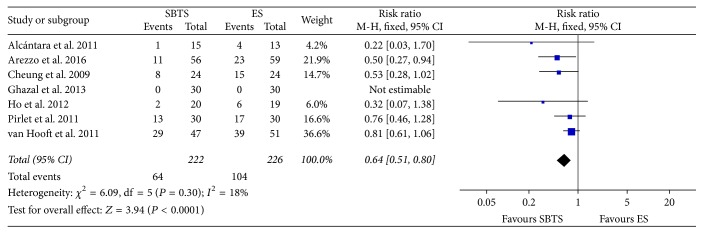
Meta-analysis of stoma rates using fixed-effect Mantel-Haenszel models. Risk ratio shown with 95% confidence intervals. ES: emergency surgery; SBTS: stent as a bridge to surgery.

As a result, stoma rate (Figure 6) was significantly reduced within the SBTS group (28.89%, 64 of 222 patients), as opposed to 46.02% (104 of 226) within the ES group (RR 0.64, 95% CI 0.51 to 0.8, *P* value < 0.0001). Heterogeneity was contained for this outcome (*I*^2^ = 18%).

**Figure 7:**
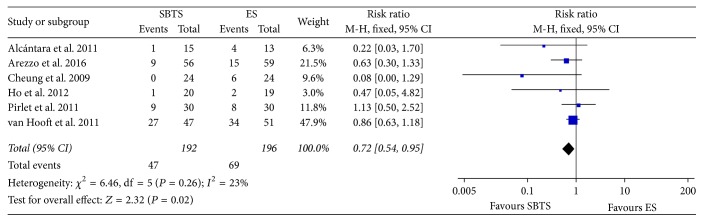
Meta-analysis of stoma rates at latest follow-up using fixed-effect Mantel-Haenszel models. Risk ratio shown with 95% confidence intervals. ES: emergency surgery; SBTS: stent as a bridge to surgery.

The necessity of a long-term stoma was reported in six RCTs as the impossibility of reverting the stoma itself or as the presence of the stoma at the latest follow-up. As shown in Figure 7, 47 of 192 patients (24.48%) within the stent group had a long-term stoma, as compared to 69 of 196 (35.2%) in the emergency surgery group (RR 0.72, 95% CI 0.54 to 0.95, *P* value < 0.02).

### 3.2. Secondary Outcomes

#### 3.2.1. Anastomotic Leak

**Figure 8:**
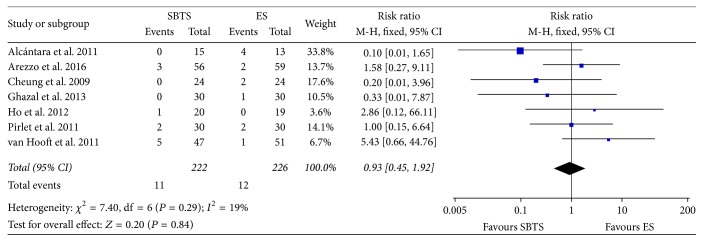
Meta-analysis of anastomotic leak rates using fixed-effect Mantel-Haenszel models. Risk ratio shown with 95% confidence intervals. ES: emergency surgery; SBTS: stent as a bridge to surgery.

Data regarding the incidence of anastomotic leak was reported in every included study. Meta-analysis did not show significant difference between the two groups, as evident in Figure 8. Heterogeneity was low (*I*^2^ = 19%); therefore the results for the fixed-effect model were considered (RR 0.93, 95% CI 0.45 to 1.92, *P* value 0.84).

#### 3.2.2. Infectious Complications

**Figure 9:**
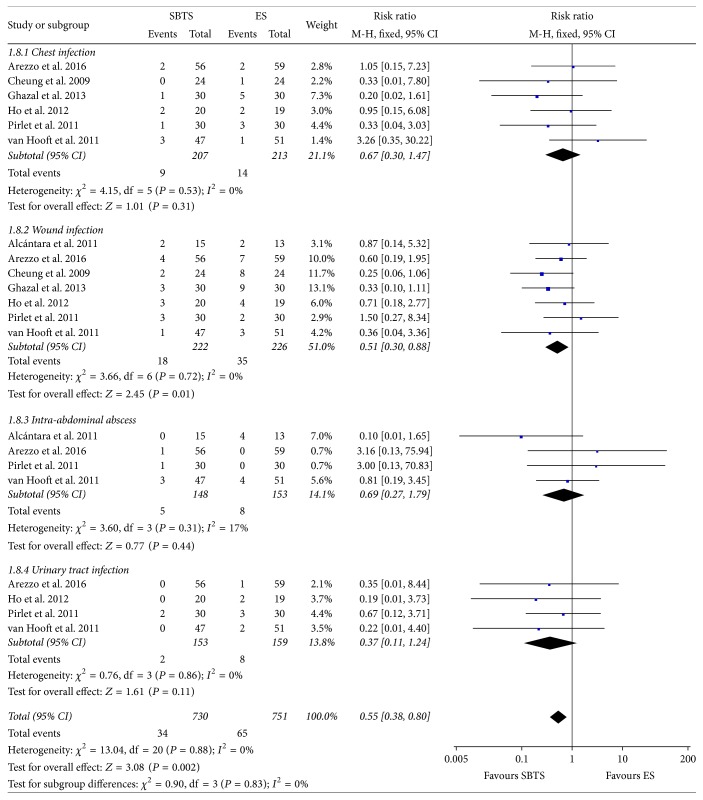
Meta-analysis of infectious complication rates using fixed-effect Mantel-Haenszel models. Risk ratio shown with 95% confidence intervals. ES: emergency surgery; SBTS: stent as a bridge to surgery.

Information regarding chest infection was reported in six studies; data on wound infection was indicated in all RCTs considered, whereas data regarding intra-abdominal abscess and urinary tract infection was available in only four studies (Figure 9).

In particular, a wound infection was found in 8.11% of the patients within the SBTS group and in 15.49% of patients receiving ES; this difference reached statistical significance (RR 0.51, 95% CI 0.30 to 0.88, *P* value 0.01). No heterogeneity was evident for this outcome (*I*^2^ = 0%) and fixed-effect model was used.

### 3.3. Stent Placement Success and Complications

Technical success was defined as correct positioning of the stent, performed either endoscopically or under radiologically guidance, whereas clinical success was defined as clinical evidence of intestinal transit or passage of flatus or stools at different time points.

In general, technical success was reported in 175 of 222 patients (78.83%) and clinical success was achieved in 75.23% of the cases. Perforation occurred in 13 patients (5.86%), while silent perforation was reported at microscopic examination in two of the considered studies [12, 13] and was found in 11 out of 77 cases (14%).

## 4. Discussion

Emergency surgical management of MBO is related to increased mortality and morbidity [3, 6], as compared to the elective setting. The placement of metallic stent in order to achieve bowel decompression could potentially allow the conversion of emergency interventions to elective/scheduled surgery and reduce the necessity of stoma formation.

Several RCTs have been conducted on this topic and led to contrasting results. Three of the considered studied were stopped prematurely. In particular, Alcántara et al. [7] reported a significant increase in the anastomotic leak rate within the ES group: 0% in the SBTS group, as compared to 30.7% in the emergency surgery group. In contrast, van Hooft et al. [13] found an increase in the absolute risk of 30-day morbidity within the SBTS group after interim analysis, with the SBTS group suffering a perforation rate equal to 13%. Pirlet et al. [12] found a relevant high number of stent-related complications, reporting 53% of technical failure and two cases of stent perforation in the SBTS group, failing to report a diminished stoma rate.

Both van Hooft et al.'s and the Pirlet et al.'s trial were multicentre RCT. The high incidence of stent-related complications might be explained by the heterogeneity in the experience of the operator endoscopists and by the high number of complete large bowel obstructions encountered. Complete obstruction was related to increased risk of complication in a retrospective study conducted by Small et al. [22]: this could be considered as a possible physiopathologic explanation to the high variability in stent-related complications among the considered studies.

The anatomical site of malignant obstruction was not reported in two of the studies considered in the current meta-analysis [9, 13]; moreover, a significant bias arising from the variable classification scheme of the tumor site is evident. Small et al. [22] reported that complications related to stents placed in the rectosigmoid colon were present in 33% of the cases. The different anatomical sites of the occlusion might be related to diverse outcomes and could be a further element of the heterogeneous results reported by different authors.

Pirlet et al. [12] described an uncomplicated postoperative course for the patients who underwent endoscopic stent placement with both technical and clinical success. A more profound understanding of the risk factors for the development of complications related to stent placement as a bridge to surgery is a key element in order to establish appropriate indications to SBTS or ES in different subgroups of patients.

Interestingly, Arezzo et al. [8] reported a high dropout rate from the trial associated with a wrong diagnosis: 20 of the 144 patients who were initially randomized (13.9%) were wrongly diagnosed at the abdominal CT scan. Similarly, van Hooft et al. [13] reported diagnostic difficulties in their trial: four patients out of the 47 initially randomized to SBTS (8.5%) were suspected to have a benign lesion at endoscopy and did not receive a stent.

As a result, stent technical and clinical success rate were 78.83% and 75.23% at the current meta-analysis, respectively, and the perforation rate was 5.89%. These results were similar to those described by Tan et al. [17].

The current meta-analysis did not show any difference between the SBTS group and the ES group in terms of in-hospital mortality. These results confirm the findings from previous studies [17, 23–26] and underline how the use of stenting techniques as a bridge to surgery might be considered a safe approach in the short term.

The incidence of postoperative complications appeared to be reduced within the SBTS group; the bias arising from various definitions of postoperative complication in different studies should be taken into consideration. The Clavien-Dindo classification [27] defines postoperative complication as every deviation from expected postsurgical course; in Arezzo et al.'s and van Hooft et al.'s trials [8, 13], the incidence of grade I complication, therefore not requiring intervention, was equal to 33.33% and 40% of the total number of complications. The results of this meta-analysis are in line with the findings of Zhang et al. [24].

Infectious complications overall appear to be reduced in the SBTS group, in particular with regard to surgical site infections, confirming that an emergency setting increases the risk of wound infection [28, 29].

Overall, the placement of metallic stents as a bridge to surgery appears to reduce short-term adverse outcome after surgery. A possible explanation of this phenomenon resides in the stabilization of the patient prior to surgery, as fluid resuscitation, correction of electrolyte imbalances, and medical work-up can be performed in the time window gained with the stent insertion. Moreover, an emergency intervention often represents a challenge in the acute setting for both the surgeon, who faces technical difficulties, and the anesthetist, who manages patients with depleted physiological reserve.

On the oncologic point of view, the time window created with the stenting might be useful for staging purposes.

As reported previously, planned surgery after the placement of a stent appeared to reduce the overall stoma rate in the current study [15, 17]; in contrast with preceding meta-analyses, our result showed a reduced long-term necessity of stoma within the SBTS group. The creation of a stoma has a relevant effect on both morbidity and quality of life [30, 31]. After stoma creation for colorectal cancer, the percentage of “problematic stoma” can be as high as 30% [32]. Therefore, this element should be considered when a decision regarding the indication to SBTS versus emergency surgery is taken in the acute setting by the surgical team.

In concordance with previous studies [15, 17], this meta-analysis did not find significant difference in the rate of primary anastomosis between the two groups; however, the trend relative to the rate of successful primary anastomosis appeared to confirm the role of stenting as a facilitator of one-stage surgical interventions. The incidence of anastomotic leak, as reported elsewhere [15, 17, 25], did not show statistical difference between SBTS and ES.

The major limitation of the current study resides in the high variability of local protocols, resulting in different surgical approaches, and of operator experience, in particular for the endoscopic stent placement. Furthermore, we found a peculiar difficulty in analyzing outcomes represented by continuous variables, for example, length of stay and duration of surgery, as a result of the elevate heterogeneity in the modality of reporting this data. In fact, some of the considered studies used median and range or interquartile ranges, possibly because of skewness of data, and some used means and standard deviation [33].

Information regarding costs for the two different treatments was available only for two studies [7, 11].

Long-term outcomes after stent placement were considered in four RCTs [7–9, 34]. Despite showing higher recurrence rates after stenting as a bridge to surgery, these studies did not report significant differences in overall survival and progression-free survival. Tumor perforation related to stent insertion was associated with lower survival rates in a single RCT [34]. However, the small number of patients considered and the relatively short follow-up period warrant caution while making inference to reach definitive conclusions on this issue. These elements are reflected in the guidelines of the ESGE [35], in which stent as a bridge to surgery is not recommended as a standard treatment for MBO, as it may be related to an increased oncologic risk without a counter-reduction in postoperative mortality; SBTS can be considered a valuable alternative to surgery in case of patients with high-surgical risk. In this sense, further evidence by large RCT is needed.

The lack of objective data regarding quality of life in this context, for example, validated questionnaires and performance status records, represents a major impairment to a thorough understanding of long-term outcomes for this peculiar group of patients. It is recognised that, in general, only a small proportion of patients receive stoma reversal as a second-time operation; SBTS is correlated with a lower stoma formation rate as compared to primary surgery and this might lead to a higher quality of life.

Current consensus exists on the assertion that metallic stents for the management MBO should be inserted only in centres where highly experienced endoscopists are available [14, 35, 36].

Further randomized controlled trials are needed in order to obtain additional evidence. Considering the substantial cost of the stenting device itself [7, 11], particular attention to the economic aspect of the management of MBO with stenting as a bridge to surgery as opposed to emergency would be beneficial in future trials. Furthermore, more accurate evidence regarding the long-term outcomes after SBTS versus emergency surgery, including quality of life and oncologic outcomes, is required in order to acquire a detailed perspective on the management of malignant large bowel obstruction.

## 5. Conclusion

The current meta-analysis showed a lower incidence of postoperative complications and decreased stoma rate and wound infection rate in the SBTS group, as compared to emergency surgery. Stenting as a bridge to surgery can be considered a safe procedure in expert hands.

Further randomized controlled trials are needed in order to perform accurate cost analysis and study long-term outcomes.

## Figures and Tables

**Table 1:** Details of randomized clinical trials included in the meta-analysis. ES: emergency surgery; IOCL: intraoperative colonic lavage; NI: not indicated; RCT: randomized controlled trial; SBTS: stent as a bridge to surgery.

Reference	Year (duration)	Type of study (*N* of centres)	Tumor site (SBTS)	Tumor site (ES)	Intervention	*N* of patients (SBTS : ES)	Type of stent	Stent to surgery time
Alcántara et al.	2011 (February 2004 to December 2006)	RCT (1)	Rectosigmoid (1) Sigmoid (11) Descending (1) Splenic flexure (2)	Rectosigmoid (3) Sigmoid (4) Descending (2) Splenic flexure (4)	Stent placement followed by planned 1-stage open surgery versus emergency 1-stage open surgery and IOCL	15 : 13	Wallstent^R^	5–7 days

Arezzo et al.	2016 (March 2008 to November 2015)	RCT (5)	Rectosigmoid (NI) Sigmoid (8) Descending (43) Splenic flexure (5)	Rectosigmoid (NI) Sigmoid (12) Descending (34) Splenic flexure (13)	Stent placement followed by planned open or laparoscopic surgery versus emergency surgery	56 : 59	Not indicated	3–8 days

Cheung et al.	2009 (January 2002 to May 2005)	RCT (1)	Not reported	Not reported	Stent placement followed by planned laparoscopic surgery versus emergency open surgery	24 : 24	Wallstent^R^	<2 weeks

Ghazal et al.	2013 (January 2009 to May 2012)	RCT (1)	Rectosigmoid (12) Sigmoid (14) Descending (4) Splenic flexure (0)	Rectosigmoid (10) Sigmoid (17) Descending (3) Splenic flexure (0)	Stent placement followed by planned 1-stage open surgery versus emergency 1-stage total abdominal colectomy and ileorectal anastomosis	30 : 30	Not indicated	7–10 days

Ho et al.	2012 (October 2004 to February 2008)	RCT (1)	Rectosigmoid (5) Sigmoid (10) Descending (3) Splenic flexure (2)	Rectosigmoid (3) Sigmoid (8) Descending (6) Splenic flexure (2)	Stent placement followed by open surgery versus emergency surgery	20 : 19	Wallflex^R^	1-2 weeks

Pirlet et al.	2011 (December 2002 to October 2006)	RCT (9)	Rectosigmoid (8) Sigmoid (15) Descending (6) Splenic flexure (0) Not available (1)	Rectosigmoid (7) Sigmoid (18) Descending (2) Splenic flexure (3) Not available (0)	Stent placement followed by open surgery versus emergency open surgery	30 : 30	Nitinol	Not reported

van Hooft et al.	2011 (March 2007 to August 2009)	RCT (25)	Not reported	Not reported	Stent placement followed by open surgery versus emergency open surgery	47 : 51	Wallstent^R^ Wallflex^R^	<4 weeks

**Table 2:** Outcomes of the studies included in the meta-analysis.

	Mortality	Postoperative complication	Primary anastomosis	Successful primary anastomosis	Stoma rate	Permanent/last f-u stoma	Anastomotic leak	Infectious complication
Alcantara et al.	+	+	+	+	+	+	+	
Arezzo et al.	+	+	+	+	+	+	+	+
Cheung et al.	+	+	+	+	+	+	+	
Ghazal et al.	+	+	+	+	+		+	
Ho et al.	+	+	+	+	+	+	+	
Pirlet et al.	+	+	+	+	+	+	+	+
van Hooft et al.	+	+	+	+	+	+	+	+

**Table 3:** Risk of bias summary. +: low risk of bias; ±: unclear risk of bias; −: high risk of bias.

	Random sequence generation	Allocation concealment	Blinding of participants and personnel	Blinding of outcome assessment	Incomplete outcome data	Selective reporting	Other bias
Alcantara et al.	+	+	±	−	+	+	+
Arezzo et al.	+	+	+	+	+	+	+
Cheung et al.	+	+	±	−	+	+	+
Ghazal et al.	+	+	±	−	+	+	+
Ho et al.	+	+	±	−	+	+	+
Pirlet et al.	+	+	±	−	+	+	+
van Hooft et al.	+	+	+	+	+	+	+

**Table 4:** Main findings for the studies included in the meta-analysis. ES: emergency surgery; FFP: fresh frozen plasma; RCT: randomized controlled trial; SBTS: stent as a bridge to surgery; SSI: surgical site infection.

Reference	Significant difference	No significant difference	Notes
Alcantara et al.	SBTS: reduced anastomotic leak rate and overall morbidity for SBTS	Mortality, median hospital stay, SSI	Trial stopped prematurely

Arezzo et al.	SBTS: decreased stoma rate, increased total length of stay	Morbidity, median operative time, oncologic outcome at 36 months	

Cheung et al.	SBTS: reduced anastomotic leak rate and stoma rate, increased rate of one-stage procedure	Median cumulative hospital stay, chest infection, intra-abdominal sepsis	

Ghazal et al.	SBTS: reduced mean operative time, necessity of blood and FFP transfusion, SSI	Median total length of stay	

Ho et al.	SBTS: reduced need for intraoperative bowel decompression	Mortality, morbidity, median operative time, medial total length of stay	

Pirlet et al.		In-hospital mortality, morbidity, stoma rate, anastomotic leak	Trial stopped prematurely

van Hooft et al.	SBTS: reduced initial stoma rate but increased stoma-related complications and increased morbidity at 30 days (interim analysis)	Global health status, mortality	Trial stopped prematurely
